# A comparative performance evaluation of 12 HIV-1 viral load testing asssays: advancing the clinical application of HIV-1 nucleic acid testing in China

**DOI:** 10.1128/spectrum.03218-24

**Published:** 2025-05-23

**Authors:** Yu Wang, Pinliang Pan, Wenge Xing, Xin Zhang, Boxue Han, Cong Jin

**Affiliations:** 1 National Key Laboratory of Intelligent Tracking and Forecasting for Infectious Diseases, National Center for AIDS/STD Control and Prevention, Chinese Center for Disease Control and Prevention 12415, Beijing, China; Penn State College of Medicine, Hershey, Pennsylvania, USA

**Keywords:** HIV-1 viral load testing, performance evaluation, nucleic acid testing, low-level viremia, point-of-care testing

## Abstract

HIV-1 viral load testing serves as a critical tool for monitoring antiretroviral therapy efficacy and detecting acute HIV-1 infections. This study evaluated the precision, linearity, and clinical performance of 12 commonly used HIV-1 viral load testing kits in China (including seven domestic and five imported kits). Precision analysis revealed that 11 kits achieved intra-assay coefficients of variation (CV%) below 5% for high and medium concentration standards at 10,000 and 1,000 IU/mL, respectively, while only 4 kits maintained comparable precision at low concentration standards (100 IU/mL). All kits showed excellent linear performance across their dynamic ranges (R² = 0.9924–0.9999) and effectively covered the major HIV-1 subtypes circulating in China. Correlation analysis of clinical samples showed strong associations (R² >0.70) in 49 out of 66 kit-pair comparisons, with Bland-Altman agreement analysis confirming good consistency between different kits. Notably, domestic Kit B exhibited minimal deviation (0.004 log10 IU/mL) compared to leading imported assays. Point-of-care testing kits demonstrated performance comparable to laboratory-based systems. However, observed variability in low viral load quantification highlights the need for improved precision to meet stringent clinical requirements for viral suppression monitoring at 50 copies/mL. These findings validate the reliability of domestic kits for clinical application while suggesting further enhancement in low-level viremia detection capabilities.

**IMPORTANCE:**

HIV-1 viral load (VL) testing is extremely important in monitoring the efficacy of antiretroviral therapy and diagnosing acute HIV-1 infections. In China, the growing demand for nucleic acid testing (NAT) and rapid development of HIV-1 NAT technology have led to the approval of 15 HIV-1 VL kits by 2024, but their performance remains understudied. This study comprehensively evaluates 12 widely used HIV-1 VL kits (seven domestic and five imported) in China, demonstrating high precision, linearity, and clinical accuracy across prevalent HIV-1 subtypes in China. Notably, domestic kits exhibited a strong correlation with imported kits, validating their reliability for clinical use. However, variability in low VL quantification highlights challenges in meeting the stringent suppression threshold of 50 copies/mL. These findings provide evidence for optimizing HIV-1 NAT strategies, supporting the adoption of cost-effective domestic kits, and guiding future improvements for low-level viremia monitoring.

## INTRODUCTION

HIV-1 viral load (VL) testing plays a critical role in monitoring the efficacy of antiretroviral therapy (ART) and diagnosing HIV-1 infections (1, 2). Particularly in the diagnosis of HIV infection, HIV-1 VL testing provides higher sensitivity and specificity compared to serological testing and can shorten the detection window period to approximately 2 weeks. These features facilitate the identification of acute HIV-1 infections (3). In recent years, advancements in HIV-1 nucleic acid testing (NAT) technology have enhanced the sensitivity and accuracy of HIV-1 VL testing, thereby further improving its clinical application in the diagnosis and treatment monitoring of HIV-1 infections (4–6).

Over the past decade, with the increasing demand for HIV-1 nucleic acid testing, the number of HIV-1 NAT laboratories in China has grown rapidly. By the end of 2024, more than 800 laboratories across the country were equipped to perform HIV-1 NAT, with their distribution expanding from the provincial level to prefectural and county levels in regions with high HIV prevalence (7). On the other hand, the development of HIV-1 NAT technology in China has progressed rapidly. By the end of 2024, the National Medical Products Administration had approved 15 HIV-1 VL testing kits, including 10 domestically produced kits and 5 imported kits (https://www.nmpa.gov.cn/datasearch/home-index.html#category=ylqx). These kits cover various detection principles, such as quantitative real-time PCR and transcription-mediated amplification, and can be used with large-scale instruments or point-of-care testing devices. The rapid development of HIV-1 VL testing kits has, to some extent, met the growing demand for HIV-1 NAT in China. However, there is currently no study that systematically analyzes and evaluates the testing performance of these kits. Particularly, given that the thresholds for assessing ART success and achieving viral suppression in China are set at 200 copies/mL and 50 copies/mL (8), respectively, the precision and accuracy of HIV-1 VL testing kits in quantifying low viral load samples have become increasingly critical.

To learn the testing performance of mainstream HIV-1 VL testing kits in the Chinese market, particularly their performance in detecting low viral load samples, we conducted a comparative analysis of the testing performance of the 12 most widely used HIV-1 VL testing kits based on data from the external quality assessment organized by the National HIV Reference Laboratory in 2024. The basic information of these 12 HIV-1 VL testing kits is listed in Table S1. In this study, we evaluated the precision and linearity of these 12 kits at both high and low viral load levels. Additionally, we analyzed the correlation and agreement of quantitative results between each pair of kits to explore their practical performance in clinical scenarios and the comparability of results, thereby assessing whether these kits can adequately meet the clinical application needs of HIV-1 NAT.

## MATERIALS AND METHODS

### Methodological evaluation

According to the guidelines on evaluating the performance of testing kits (9–14), the precision, linearity, and clinical performance of the 12 mainstream HIV-1 VL kits were evaluated, as well as the correlation and agreement between each pair of kits.

### Precision analysis

Three concentrations of standards—high (10,000 IU/mL), medium (1,000 IU/mL), and low (100 IU/mL)—were prepared by diluting the National Standard for HIV-1 RNA quantification (130,000 IU/mL; National Institutes for Food and Drug Control, Beijing, China) with HIV-1-negative plasma. In each trial run, the high-, medium-, and low-concentration standards were repeatedly tested five times, and a total of two independent runs were conducted. To evaluate the precision of the assay, the intra-run coefficient of variation (CV%) for each concentration was calculated using the equation below (9–13).


$$Intra-run\;CV\%=\frac{\sqrt{\frac{Var\;.run1+Var\;.run2}{2}}}{Mean}$$

### Linearity analysis

The linearity of HIV-1 VL kits was evaluated using five samples spanning the limit of quantitation range for each assay, typically ranging from 10² to 10⁶ copies/mL. These five samples were prepared by fivefold serial dilution of an HIV-1 subtype CRF_07BC sample (5.7 log10 IU/mL), as determined by the COBAS TaqMan HIV-1 Test v2.0 assay (Roche, Switzerland). For each HIV-1 VL kit, the five samples were tested in triplicate, and a standard curve was constructed based on the mean results. The linear regression equation and linear correlation coefficient were then calculated for each kit (9–12, 14).

### Clinical performance

The sensitivity of HIV-1 VL kits was evaluated by 28 HIV-1 positive samples, including 20 samples with VL ≥5,000 IU/mL and 8 samples with VL <5,000 IU/ml (5 samples with VL between 1,000 and 5,000 IU/mL, 2 samples with VL between 200 and 1,000, and 1 sample with VL <200), as determined by the COBAS TaqMan HIV-1 Test v2.0. These samples covered the four most prevalent HIV-1 subtypes in China: CRF_07BC, CRF_08BC, CRF_01AE, and CRF_5501B. The specificity was evaluated by 29 HIV-1-negative samples, including three hepatitis C virus-positive samples, three hepatitis B virus-positive samples, and three Treponema pallidum-positive samples (9, 10).

### Statistical analysis

For statistical analysis, all HIV-1 RNA values expressed in copies/mL were converted to IU/mL and then transformed to log_10_ IU/mL. The correlation between each pair of kits was assessed using linear regression analysis, and the agreement between each pair of kits was evaluated using Bland-Altman analysis. The results were analyzed and plotted with the GraphPad Prism version 8.0 software (Boston, MA, USA).

## RESULTS

### Precision analysis of 12 HIV-1 VL kits at different standard concentrations

When analyzing high-concentration standards, six kits had an intra-run CV% <2%, including three internal standard quantification (ISQ) kits and three external standard quantification (ESQ) kits (Table 1). Five kits showed intra-run CV% between 2% and 5%, comprising four ISQ kits and one ESQ kit. Only one ESQ kit (Kit D) exhibited an intra-run CV% >5% at high concentration. For medium-concentration standards, only one ESQ kit (Kit J) achieved an intra-run CV% <2%, while the other 10 kits (excluding Kit D) all demonstrated intra-run CV% values within the 2%–5% range. For low-concentration standards, intra-run CV% could only be calculated for nine kits. Among these kits, four kits (three ISQ and one ESQ) showed intra-run CV% values between 2% and 5%, while the other five kits had intra-run CV% >5%.

**TABLE 1:** Precision analysis of 12 HIV-1 VL kits at different concentrations^*a*^

Kit	Concentration (IU/mL)	Mean (IU/mL)	Var. [(IU/mL)^2^]		Intra-run CV (%)
			Run 1	Run 2	
A_P_^*d*^	10,000	3.61	0.0012	0.0021	1.06
	1,000	2.87	0.0050	0.0105	2.80
	100	NC^*b*^	NC	NC	NC
B	10,000	3.43	0.0033	0.0083	2.23
	1,000	2.71	0.0045	0.0206	4.14
	100	2.37	0.0052	0.0156	4.32
C^*d*^	10,000	3.92	0.0016	0.0015	0.10
	1,000	2.28	0.0021	0.0311	3.93
	100	2.61	0.0111	0.0122	4.14
D	10,000	3.18	0.047	0.047	6.80
	1,000	ND^*c*^	ND	ND	ND
	100	ND	ND	ND	ND
E^*d*^	10,000	3.99	0.0153	0.0045	2.49
	1,000	3.07	0.0029	0.0134	2.94
	100	2.40	0.0016	0.0095	3.11
F^*d*^	10,000	4.03	0.0141	0.0098	2.72
	1,000	2.99	0.0169	0.0064	3.61
	100	2.14	0.0078	0.0107	4.49
G^*d*^	10,000	3.93	0.0020	0.0043	1.44
	1,000	2.84	0.0054	0.0102	3.11
	100	NC	NC	NC	NC
H	10,000	4.03	0.0036	0.0027	1.40
	1,000	3.05	0.0086	0.0092	3.09
	100	1.99	0.2067	0.1755	21.92
I	10,000	3.77	0.0015	0.0018	1.08
	1,000	2.76	0.0053	0.0017	2.14
	100	1.74	0.0505	0.0381	12.07
J	10,000	3.46	0.0141	0.0216	3.86
	1,000	2.83	0.0016	0.0018	1.46
	100	2.06	0.0476	0.0143	8.56
K	10,000	3.94	0.0164	0.0022	2.44
	1,000	3.48	0.0157	0.0083	3.15
	100	2.55	0.0304	0.0100	5.58
L	10,000	3.88	0.0053	0.0045	1.81
	1,000	2.96	0.0029	0.0059	2.24
	100	1.83	0.0494	0.0571	12.59

^*a*^ P: POCT, point of care test.

^*b*^ NC, not all five tests in a run could provide VL values; some results detected HIV-1 RNA, but the VL value was lower than LLOQ.

^*c*^ ND, not all five tests in a run could detect HIV-1 RNA.

^*d*^ Imported HIV-1 VL kit.

Notably, the point-of-care (POC) kit A_P_^*^ displayed intra-run CV% values of <2% and 2%–5% for high and medium concentrations, respectively. However, its intra-run CV% at low concentration could not be determined due to measurements falling below the lower limit of quantification.

### All 12 HIV-1 VL kits exhibited excellent linearity within their quantitative ranges

**Fig 1:**
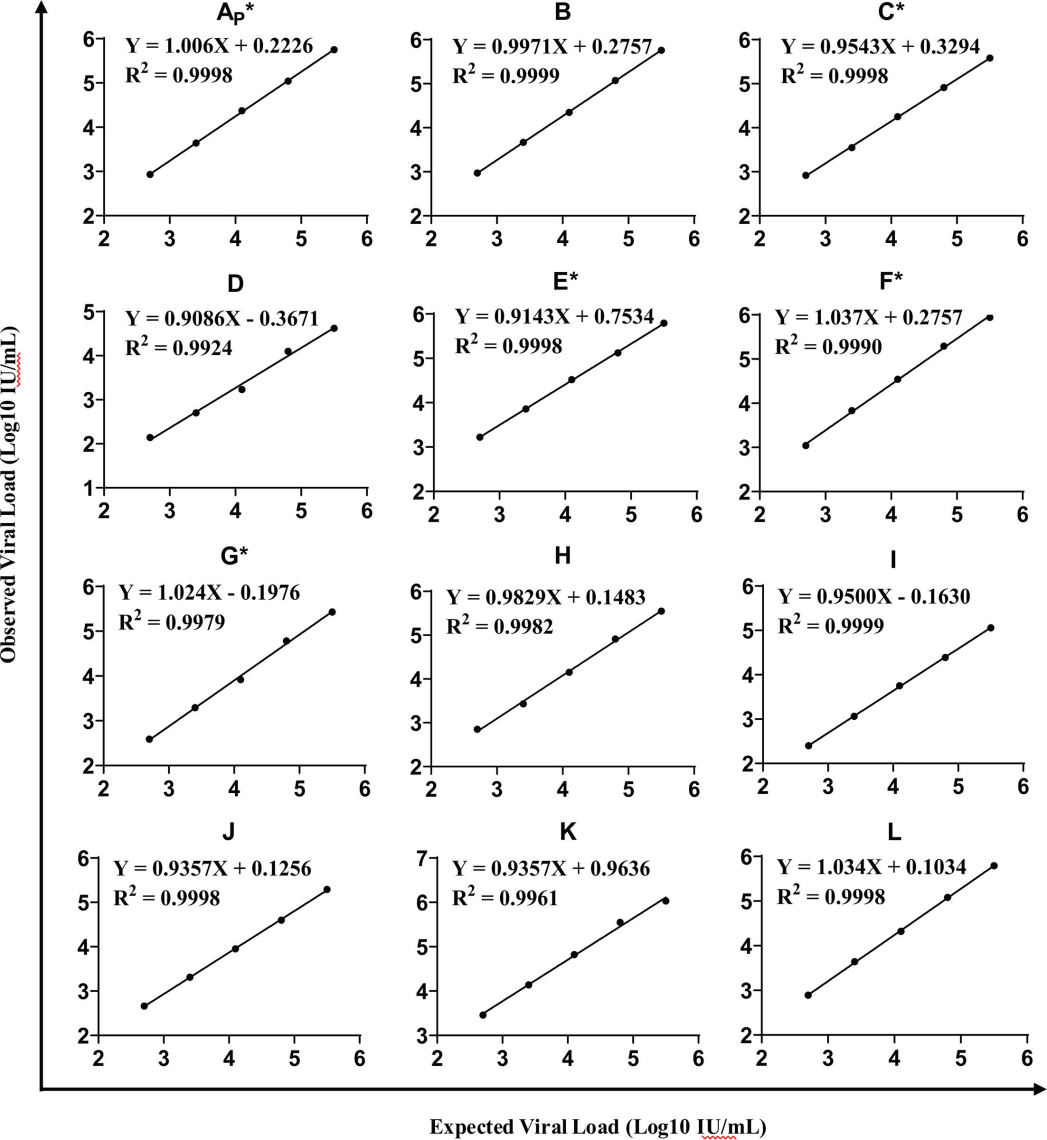
Linear regression plots of 12 HIV-1 VL kits. Viral load testing results are plotted against the expected value of diluted standards. The solid line represents the fitted linear trend.

The linear regression coefficients for all the 12 HIV-1 VL kits exceeded 0.99, ranging from 0.9924 to 0.9999 (Fig. 1), confirming that all these kits exhibited good linear performance across their quantitative measurement ranges.

### All 12 HIV-1 VL kits demonstrated excellent clinical performance

The sensitivity and specificity of all 12 HIV-1 VL kits were 100%. For HIV-1-positive samples, these kits can effectively identify 20 high-concentration samples and eight low-concentration samples and were capable of detecting four prevalent HIV-1 subtypes in China. For HIV-1-negative samples, including nine potentially interfering samples, all 12 kits produced negative results, demonstrating excellent specificity.

### Strong correlation was observed among the 12 HIV-1 VL kits

**Fig 2:**
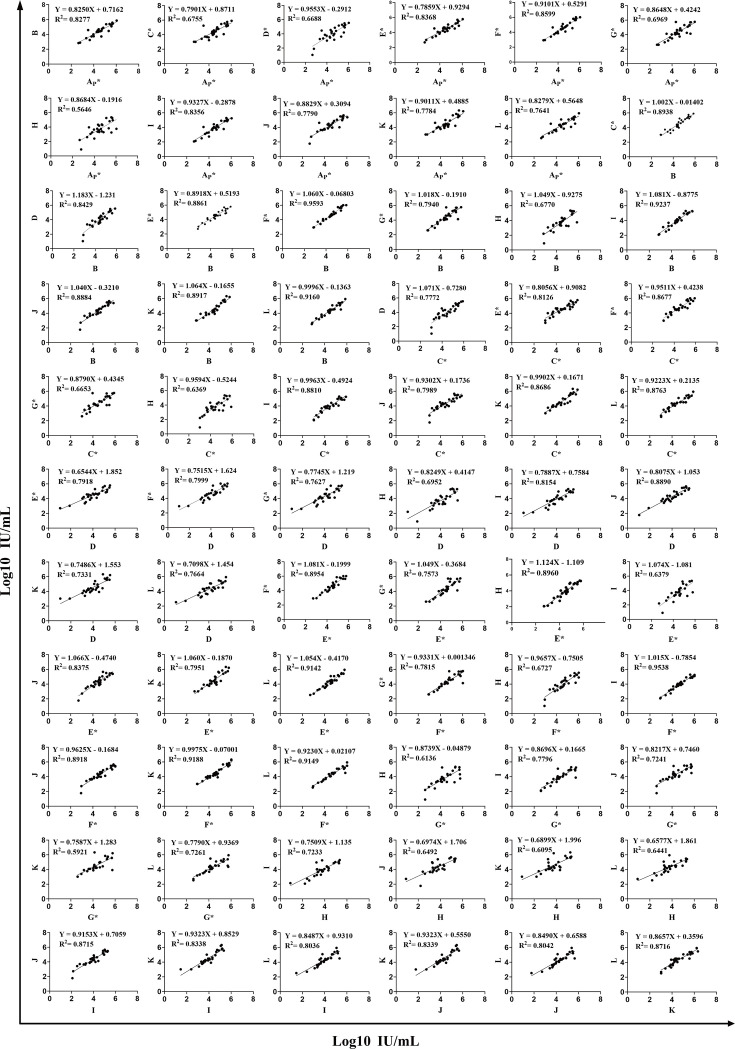
Correlation analysis between test results of 12 HIV-1 VL kits. Each black dot represents the test result of a positive sample, and the solid line represents the fitted linear trend. R^2^ and conversion formulae for the correlation analysis of the paired kits are labeled on each graph.

Quantitative results from each pair of kits were compared using linear regression (Fig. 2). Among the 66 comparisons, seven pairs showed statistically significant strong linear correlations with the correlation coefficient (R^2^) >0.90. Among these seven comparisons, two pairs were between domestic kits, and five pairs were between domestic and imported kits. The highest correlation (R^2^ = 0.9593) was observed between domestic kit B and an imported kit F*. The remaining comparisons showed that there were 26 pairs with R^2^ values of 0.80–0.90 and 18 pairs with R^2^ values of 0.70–0.80. The lowest correlation (R^2^ = 0.5646) was observed between the POC kit A_P_* and domestic kit H. The linear regression equations and R^2^ values are summarized in Table S2.

### High agreement was observed among the 12 HIV-1 viral load assays

**Fig 3:**
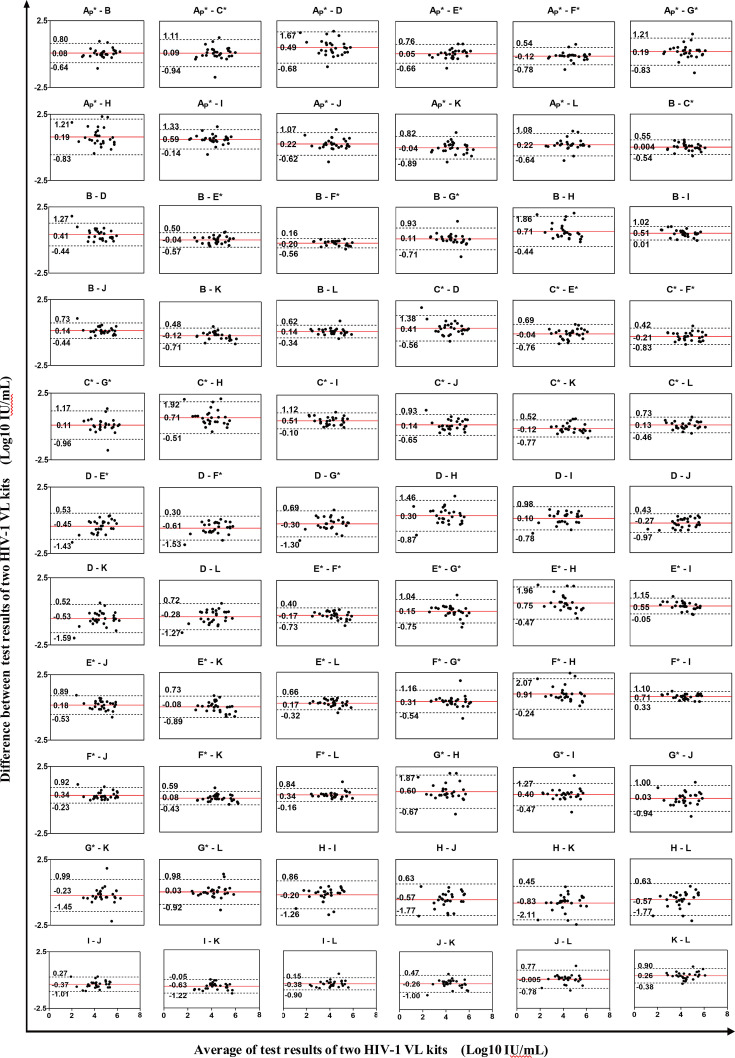
Bland-Altman agreement analysis of 12 HIV-1 VL kits. Each black circle represents the test result of a positive sample. The red solid line indicates the mean bias of paired kits, and the two black dashed lines indicate the upper and lower limits of agreement (±1.96 SD).

Pairwise comparisons of the 66 kits revealed a bias range of 0.0043–0.9107 log10 IU/mL (Fig. 3). The minimal bias was shown between domestic Kit B and imported Kit C*, while the maximal bias was observed between domestic Kit H and imported Kit F*. Notably, domestic Kit B demonstrated excellent agreement with imported Kits C* and E*, with biases of 0.004 and 0.04 log10 IU/mL, respectively, and limits of agreement (LOA) intervals of 1.09 and 1.07. The point-of-care (POC) kit A_P_^*^ also showed strong agreement with both the domestic Kit B (bias = 0.0811, LOA interval = 1.44) and imported Kit E* (bias = 0.0461, LOA interval = 1.42). Complete agreement analysis data are presented in Table S2.

## DISCUSSION

This study conducted a comprehensive and systematic comparison of 12 mainstream HIV-1 VL kits used in China. The results demonstrated strong correlations among most of the tested assays. Based on correlation data, conversion formulas between different kits were calculated, which will facilitate the conversion of test results across different kits and provide comparable HIV-1 VL data for monitoring the ART effect on HIV-infected individuals.

For many years, HIV-1 VL testing in China has primarily relied on imported kits, with their high cost limiting implementation in resource-limited areas. In recent years, the development of domestic assays has expanded options for HIV-1 VL testing. This study demonstrates that the results of the commonly used domestic kits in China are highly comparable and correlated with the imported kits, particularly for the most commonly used domestic kit B, as the R² and agreement bias with imported kits reached 0.9593 and 0.004, respectively, indicating that domestic kits can serve as a reliable alternative. However, while overall performance was comparable, we observed suboptimal correlation and agreement between some kits, with imported kits and dual-target kits generally yielding higher quantitative values than domestic and single-target assays, respectively. These differences are reasonable given that components such as enzyme types and primer/probe target regions determine varying amplification efficiencies across subtypes (15, 16). Additionally, differences in quantification algorithms and calibration standards among kits may also contribute to quantitative deviations (17, 18). Notably, the rapid development of point-of-care testing (POCT) technology has made HIV-1 NAT feasible in resource-limited settings. This study found that POCT kits exhibited comparable performance to conventional laboratory-based kits, providing a new option for resource-limited areas and expanding detection approaches for acute HIV-1 infection (19, 20).

In recent years, the precise and accurate quantification of low VL samples has become increasingly critical given the growing number of HIV-1 low-level viremia (21). Accordingly, both the WHO and China have adjusted the criteria for ART success and viral suppression to 200 copies/mL and 50 copies/mL, respectively (8, 22, 23). Therefore, we selected 100 IU/mL as the low-concentration standard to assess whether the 12 HIV-1 kits could provide precise and accurate quantitative results. And our findings found that some kits showed CV% values exceeding 5% at low concentrations.

Several factors may influence low-concentration quantification performance. Firstly, inherent limitations of qPCR methodology contribute to the inaccuracy in detecting low VL. Previous studies have shown that when concentrations approach the kit’s lower limit of quantification, CV% up to 20% is considered acceptable (24). This emphasizes the necessity for multiple tests in clinical practice to comprehensively evaluate treatment efficacy in HIV-1 infections. Secondly, quantification methodology plays an important role. In this study, we found that ISQ kits generally outperformed ESQ kits in precision, primarily due to their methodology of detecting samples and standards in the same reaction well, thereby minimizing variability in extraction and amplification processes (25–27). Finally, compatibility between assay components and sample types may affect performance. We observed that kit D failed to detect some medium and low concentration standards that are prepared with virus culture materials, but performed well with the standards carried in the kits, which are prepared by virus-like particles, indicating its suboptimal performance for authentic viruses. And this finding suggests that future development of HIV-1 VL kits should use virus cultures or real clinical samples for performance evaluation and optimization to better match clinical sample characteristics.

In conclusion, while the 12 evaluated HIV-1 VL kits demonstrated generally comparable and interchangeable performance overall, some kits still require improvement in quantification precision for low VL samples to better meet the needs of ART monitoring, particularly given the current use of 50 copies/mL as the viral suppression threshold.

## SUPPLEMENTAL MATERIAL

Supplemental material spectrum.03218-24-s0001.docx Tables S1 and S2.

ASM does not own the copyrights to Supplemental Material that may be linked to, or accessed through, an article. The authors have granted ASM a non-exclusive, world-wide license to publish the Supplemental Material files. Please contact the corresponding author directly for reuse.

## References

[1] Narasimhan M, Kapila M. 2019. Implications of self-care for health service provision. Bull World Health Organ 97:76–76A. doi:10.2471/BLT.18.228890 30728611 PMC6357575

[2] Satcher Johnson A, Song R, Hall HI. 2017. Estimated HIV incidence, prevalence, and undiagnosed infections in US States and Washington, DC, 2010-2014. J Acquir Immune Defic Syndr 76:116–122. doi:10.1097/QAI.0000000000001495 28700407

[3] Hunt PW. 2017. Very early ART and persistent inflammation in treated HIV. Clin Infect Dis 64:132–133. doi:10.1093/cid/ciw697 27965300 PMC5215217

[4] Chinese Center For Disease Control And Prevention. 2020. Chapter IV. HIV-1 nucleic acid test, p 16–21. In National guideline for detection of HIV/AIDS, 2020 ed. China CDC, Beijing.

[5] World Health Organization. 2013. Chapter VII. Clinical guidance across the continuum of care: antiretroviral therapy, p 97–153. In Consolidated guidelines on the use of antiretroviral drugs for treating and preventing HIV-1 infection: recommendations for a public health approach. WHO, Geneva.

[6] Luft LM, Gill MJ, Church DL. 2011. HIV-1 viral diversity and its implications for viral load testing: review of current platforms. Int J Infect Dis 15:e661–e670. doi:10.1016/j.ijid.2011.05.013 21767972

[7] Pan P, Xue Y, Gao J, Zhu Q, Liu J, Jiang Y, Jin C. 2021. Fifteen years of the proficiency testing program for HIV-1 viral load testing laboratories in China, 2005-2019. J Clin Virol 142:104911. doi:10.1016/j.jcv.2021.104911 34332435

[8] AIDS and Hepatitis C Professional Group, Society of Infectious Diseases, Chinese Medical Association, Chinese Center for Disease Control and Prevention. 2021. Chinese guidelines for diagnosis and treatment of HIV/AIDS, 2021 ed. Zhonghua Nei Ke Za Zhi 60:1106–1128. doi:10.3760/cma.j.cn112138-20211006-00676 34856684

[9] World Health Organization. 2017. Chapter VII. Product performance specifications and associated validation and verification studies, p 37–66. In WHO prequalification: sample product dossier for a quantitative nucleic acid-based testing technology to measure HIV-1 RNA. WHO, Geneva.

[10] World Health Organization. 2017. Protocol for the laboratory evaluation of nucleic acid based HIV-1viral load testing technologies. Geneva

[11] China National Accreditation Service for Conformity Assessment. 2019. Guidance on the performance verification for molecular diagnostic procedures. Beijing

[12] China National Accreditation Service for Conformity Assessment. 2019. Guidance on the verification of quantitative measurement procedures used in the clinical chemistry. Beijing

[13] Clinical and Laboratory Standards Institute. 2006. Chapter VIII. Verification of precision performance, p 6–10. In User verification of performance for precision and trueness; approved guideline, 2nd ed. CLSI, Wayne, PA.

[14] Clinical and Laboratory Standards Institute. 2012. Chapter V. Protocols for evaluation of the limit of blank and limit of detection, p 10–26. In Evaluation of detection capability for clinical laboratory measurement procedures; approved guideline, 2nd ed. CLSI, Wayne, PA.

[15] Mor O, Gozlan Y, Wax M, Mileguir F, Rakovsky A, Noy B, Mendelson E, Levy I. 2015. Evaluation of the RealTime HIV-1, Xpert HIV-1, and Aptima HIV-1 Quant Dx assays in comparison to the NucliSens EasyQ HIV-1 v2.0 assay for quantification of HIV-1 viral load. J Clin Microbiol 53:3458–3465. doi:10.1128/JCM.01806-15 26292298 PMC4609691

[16] Shah K, Ragupathy V, Saga A, Hewlett I. 2016. High sensitivity detection of HIV-1 using two genomic targets compared with single target PCR. J Med Virol 88:1092–1097. doi:10.1002/jmv.24431 26575693

[17] Amendola A, Sberna G, Forbici F, Abbate I, Lorenzini P, Pinnetti C, Antinori A, Capobianchi MR. 2020. The dual-target approach in viral HIV-1 viremia testing: an added value to virological monitoring? PLoS One 15:e0228192. doi:10.1371/journal.pone.0228192 32023284 PMC7001951

[18] Sberna G, Sarti S, Cicalini S, Antinori A, Garbuglia AR, Amendola A. 2022. Evaluating the dual-target Aptima HIV-1 Quant Dx assay: comparison between viral loads measured with pol and LTR targets in the same samples. Microbiol Spectr 10:e0136122. doi:10.1128/spectrum.01361-22 36066258 PMC9603300

[19] Ceffa S, Luhanga R, Andreotti M, Brambilla D, Erba F, Jere H, Mancinelli S, Giuliano M, Palombi L, Marazzi MC. 2016. Comparison of the Cepheid GeneXpert and Abbott M2000 HIV-1 real time molecular assays for monitoring HIV-1 viral load and detecting HIV-1 infection. J Virol Methods 229:35–39. doi:10.1016/j.jviromet.2015.12.007 26709099

[20] Wesolowski L, Fowler W, Luo W, Sullivan V, Masciotra S, Smith T, Rossetti R, Delaney K, Oraka E, Chavez P, Ethridge S, Switzer WM, Owen SM. 2020. Evaluation of the performance of the Cepheid Xpert HIV-1 viral load assay for quantitative and diagnostic uses. J Clin Virol 122:104214. doi:10.1016/j.jcv.2019.104214 31835210 PMC11089535

[21] Zhao S, Wang W, Li S, He J, Duan W, Fang Z, Ma X, Li Z, Guo C, Wang W, Wu H, Zhang T, Huang X. 2025. The prevalence of low-level viraemia and its association with virological failure in people living with HIV: a systematic review and meta-analysis. Emerg Microbes Infect 14:2447613. doi:10.1080/22221751.2024.2447613 39727007 PMC11722027

[22] World Health Organization. 2021. Chapter III. Clinical guidelines: diagnostics and treatment monitoring, p 19–43. In Updated recommendations on HIV prevention, infant diagnosis, antiretroviral initiation and monitoring. WHO, Geneva. 33822559

[23] World Health Organization. 2023. The role of HIV viral suppression in improving individual health and reducing transmission. Geneva

[24] Armbruster DA, Pry T. 2008. Limit of blank, limit of detection and limit of quantitation. Clin Biochem Rev 29 Suppl 1:S49–S52. 18852857 PMC2556583

[25] Yun JJ. 2006. Genomic DNA functions as a universal external standard in quantitative real-time PCR. Nucleic Acids Res 34:e85–e85. doi:10.1093/nar/gkl400 16840529 PMC1524913

[26] Souazé F, Ntodou-Thomé A, Tran CY, Rostène W, Forgez P. 1996. Quantitative RT-PCR: limits and accuracy. Biotechniques 21:280–285. doi:10.2144/96212rr01 8862813

[27] Harshitha R, Arunraj DR. 2021. Real-time quantitative PCR: a tool for absolute and relative quantification. Biochem Mol Biol Educ 49:800–812. doi:10.1002/bmb.21552 34132460

