# Supplementary material for: A comparative performance evaluation of 12 HIV-1 viral load testing asssays: advancing the clinical application of HIV-1 nucleic acid testing in China
Source: Microbiol Spectr. 2025 May 23;13(7):e03218-24. doi: 10.1128/spectrum.03218-24 (PMC12210933; doi:10.1128/spectrum.03218-24)
Supplement: Supplemental material — Tables S1 and S2. [file spectrum.03218-24-s0001.docx]

Table S1. Basic information of 12 HIV-1 VL kits

| Kit | Detection principle | Target | Sample volume | LOD | LLOQ | ULOQ | Quantitative method | Market  time |
| --- | --- | --- | --- | --- | --- | --- | --- | --- |
| A_P_^*^ | qPCR | *LTR* | 1mL | 31.5IU/mL | 69IU/mL | 1.72×10^7^ IU/mL | ISQ^#^ | 2019 |
| B | qPCR | *LTR, pol* | 700μL | 30IU/mL | 100IU/mL | 1×10^7^ IU/mL | ISQ | 2019 |
| C^*^ | qPCR | *pol* | 600μL | 70IU/mL | 70IU/mL | 1.75×10^7^ IU/mL | ESQ^&^ | 2016 |
| D | qPCR | *gag* | 400μL | 50IU/mL | 200IU/mL | 1×10^9^ IU/mL | ESQ | 2017 |
| E^*^ | qPCR | *LTR, gag* | 1mL | 33IU/mL | 33.3IU/mL | 1.67×10^7^ IU/mL | ISQ | 2014 |
| F^*^ | qPCR | *LTR, gag* | 400μL | 23.6IU/mL | 33.3IU/mL | 1.67×10^7^ IU/mL | ISQ | 2020 |
| G^*^ | TMA ^a^ | *LTR, pol* | 700μL | 35IU/mL | 57IU/mL | 5.7×10^7^ IU/mL | ISQ | 2022 |
| H | qPCR | *gag* | 500μL | 85IU/mL | 85IU/mL | 1.7×10^7^ IU/mL | ESQ | 2017 |
| I | qPCR | *LTR, gag* | 400μL | 25IU/mL | 50IU/mL | 1×10^8^ IU/mL | ESQ | 2022 |
| J | qPCR | *gag, pol* | 600μL | 33IU/mL | 50IU/mL | 2×10^7^ IU/mL | ESQ | 2021 |
| K | SAT ^b^ | *pol* | 800μL | 30IU/mL | 100IU/mL | 1×10^8^ IU/mL | ISQ | 2023 |
| L | qPCR | *LTR, gag* | 200μL | 30IU/mL | 50IU/mL | 1×10^8^ IU/mL | ESQ | 2022 |

P: POCT = Point of care test

&: ESQ = External standard quantification

#: ISQ = Internal standard quantification

a: TMA = Transcription mediated amplification

b: SAT = Simultaneous amplification and testing

*: Imported HIV-1 VL kit

Table S2. Comparative evaluation of 12 HIV-1 VL kits

| Kit | Regression equation | R^2^ | Agreement | LOA^#^ | Bias | P value |
| --- | --- | --- | --- | --- | --- | --- |
| A_P_^*^ & B | y = 0.8250x + 0.7162 | 0.8277 | 25/28 (89.29%) | -0.6426, 0.8047 | 0.0810 | < 0.001 |
| A_P_^*^ & C^*^ | y = 0.7901x + 0.8711 | 0.6755 | 26/28 (92.86%) | -0.9370, 1.108 | 0.0853 | < 0.001 |
| A_P_^*^ & D | y = 0.9553x - 0.2912 | 0.6688 | 26/28 (92.86%) | -0.6798, 1.669 | 0.4946 | < 0.001 |
| A_P_^*^ & E^*^ | y = 0.7859x + 0.9294 | 0.8368 | 25/28 (89.29%) | -0.6648, 0.7569 | 0.0460 | < 0.001 |
| A_P_^*^ & F^*^ | y = 0.9101x + 0.5291 | 0.8599 | 25/28 (89.29%) | -0.7789, 0.5396 | -0.1196 | < 0.001 |
| A_P_^*^ & G^*^ | y = 0.8648x + 0.4242 | 0.6969 | 26/28 (92.86%) | -0.83, 1.214 | 0.1918 | < 0.001 |
| A_P_^*^ & H | y = 0.8684x - 0.1916 | 0.5646 | 26/28 (92.86%) | -0.5579, 2.14 | 0.7911 | < 0.001 |
| A_P_^*^ & I | y = 0.9327x - 0.2878 | 0.8356 | 26/28 (92.86%) | -0.1363, 1.325 | 0.5943 | < 0.001 |
| A_P_^*^ & J | y = 0.8829x + 0.3094 | 0.7790 | 26/28 (92.86%) | -0.621, 1.069 | 0.2239 | < 0.001 |
| A_P_^*^ & K | y = 0.9011x + 0.4885 | 0.7784 | 26/28 (92.86%) | -0.8936, 0.8179 | -0.0378 | < 0.001 |
| A_P_^*^ & L | y = 0.8279x + 0.5648 | 0.7641 | 24/28 (85.71%) | -0.6368, 1.075 | 0.2193 | < 0.001 |
| B & C^*^ | y = 1.002x - 0.01402 | 0.8938 | 27/28 (96.43%) | -0.5418, 0.5504 | 0.0043 | < 0.001 |
| B & D | y = 1.183x - 1.231 | 0.8429 | 27/28 (96.43%) | -0.4437, 1.271 | 0.4136 | < 0.001 |
| B & E^*^ | y = 0.8918x + 0.5193 | 0.8861 | 26/28 (92.86%) | -0.5686, 0.4986 | -0.035 | < 0.001 |
| B & F^*^ | y = 1.060x - 0.06803 | 0.9593 | 27/28 (96.43%) | -0.5587, 0.1573 | 0.1827 | < 0.001 |
| B & G^*^ | y = 1.018x - 0.1910 | 0.7940 | 26/28 (92.86%) | -0.7094, 0.9308 | 0.1107 | < 0.001 |
| B & H | y = 1.049x - 0.9275 | 0.6770 | 26/28 (92.86%) | -0.4376, 1.858 | 0.7100 | < 0.001 |
| B & I | y = 1.081x - 0.8775 | 0.9237 | 27/28 (96.43%) | 0.005459, 1.021 | 0.5132 | < 0.001 |
| B & J | y = 1.040x - 0.3210 | 0.8884 | 27/28 (96.43%) | -0.4431, 0.7288 | 0.1429 | < 0.001 |
| B & K | y = 1.064x - 0.1655 | 0.8917 | 26/28 (92.86%) | -0.7133, 0.4755 | -0.1189 | < 0.001 |
| B & L | y = 0.9996x - 0.1363 | 0.9160 | 26/28 (92.86%) | -0.3403, 0.6167 | 0.1382 | < 0.001 |
| C^*^ & D | y = 1.071x - 0.7280 | 0.7772 | 26/28 (92.86%) | -0.5594, 1.378 | 0.4093 | < 0.001 |
| C^*^ & E^*^ | y = 0.8056x + 0.9082 | 0.8126 | 27/28 (96.43%) | -0.7647, 0.6862 | -0.0393 | < 0.001 |
| C^*^ & F^*^ | y = 0.9511x + 0.4238 | 0.8677 | 27/28 (96.43%) | -0.7322, 0.4007 | 0.2890 | < 0.001 |
| C^*^ & G^*^ | y = 0.8790x + 0.4345 | 0.6653 | 26/28 (92.86%) | -0.9578, 1.171 | 0.1064 | < 0.001 |
| C^*^ & H | y = 0.9594x - 0.5244 | 0.6369 | 26/28 (92.86%) | -0.5102, 1.922 | 0.7057 | < 0.001 |
| C^*^ & I | y = 0.9963x - 0.4924 | 0.8810 | 27/28 (96.43%) | -0.1046, 1.122 | 0.5089 | < 0.001 |
| C^*^ & J | y = 0.9302x + 0.1736 | 0.7989 | 27/28 (96.43%) | -0.6522, 0.9293 | 0.1386 | < 0.001 |
| C^*^ & K | y = 0.9902x + 0.1671 | 0.8686 | 25/28 (89.29%) | -0.7689, 0.5225 | -0.1232 | < 0.001 |
| C^*^ & L | y = 0.9223x + 0.2135 | 0.8763 | 26/28 (92.86%) | -0.4611, 0.7290 | 0.1339 | < 0.001 |
| D & E^*^ | y = 0.6544x + 1.852 | 0.7918 | 27/28 (96.43%) | -1.429, 0.5324 | -0.4486 | < 0.001 |
| D & F^*^ | y = 0.7515x + 1.624 | 0.7999 | 26/28 (92.86%) | -1.532, 0.3032 | -0.6143 | < 0.001 |
| D & G^*^ | y = 0.7745x + 1.219 | 0.7627 | 26/28 (92.86%) | -1.295, 0.6894 | -0.3029 | < 0.001 |
| D & H | y = 0.8249x + 0.4147 | 0.6952 | 26/28 (92.86%) | -0.8715, 1.464 | 0.2964 | < 0.001 |
| D & I | y = 0.7887x + 0.7584 | 0.8154 | 27/28 (96.43%) | -0.7773, 0.9766 | 0.0996 | < 0.001 |
| D & J | y = 0.8075x + 1.053 | 0.8890 | 28/28 (100%) | -0.9716, 0.4302 | -0.2707 | < 0.001 |
| D & K | y = 0.7486x + 1.553 | 0.7331 | 26/28 (92.86%) | -1.585, 0.5201 | -0.5325 | < 0.001 |
| D & L | y = 0.7098x + 1.454 | 0.7664 | 26/28 (92.86%) | -1.268, 0.7177 | -0.2754 | < 0.001 |
| E^*^ & F^*^ | y = 1.081x - 0.1999 | 0.8954 | 27/28 (96.43%) | -0.7322, 0.4007 | 0.2890 | < 0.001 |
| E^*^ & G^*^ | y = 1.049x - 0.3684 | 0.7573 | 25/28 (89.29%) | -0.747, 1.038 | 0.1457 | < 0.001 |
| E^*^ & H | y = 1.124x - 1.109 | 0.8960 | 25/28 (89.29%) | -0.4724, 1.962 | 0.7450 | < 0.001 |
| E^*^ & I | y = 1.074x - 1.081 | 0.6379 | 25/28 (89.29%) | -0.05474, 1.151 | 0.5482 | < 0.001 |
| E^*^ & J | y = 1.066x - 0.4740 | 0.8375 | 26/28 (92.86%) | -0.532, 0.8877 | 0.1779 | < 0.001 |
| E^*^ & K | y = 1.060x - 0.1870 | 0.7951 | 27/28 (96.43%) | -0.8949, 0.7270 | -0.0839 | < 0.001 |
| E^*^ & L | y = 1.054x - 0.4170 | 0.9142 | 27/28 (96.43%) | -0.3172, 0.6636 | 0.1732 | < 0.001 |
| F^*^ & G^*^ | y = 0.9331x + 0.001346 | 0.7815 | 26/28 (92.86%) | -0.5404, 1.163 | 0.3114 | < 0.001 |
| F^*^ & H | y = 0.9657x - 0.7505 | 0.6727 | 26/28 (92.86%) | -0.2434, 2.065 | 0.9107 | < 0.001 |
| F^*^ & I | y = 1.015x - 0.7854 | 0.9538 | 27/28 (96.43%) | 0.3307, 1.097 | 0.7139 | < 0.001 |
| F^*^ & J | y = 0.9625x - 0.1684 | 0.8918 | 26/28 (92.86%) | -0.2337, 0.9209 | 0.3436 | < 0.001 |
| F^*^ & K | y = 0.9975x - 0.07001 | 0.9188 | 27/28 (96.43%) | -0.4255, 0.5891 | 0.0818 | < 0.001 |
| F^*^ & L | y = 0.9230x + 0.02107 | 0.9149 | 27/28 (96.43%) | -0.1603, 0.8382 | 0.3389 | < 0.001 |
| G^*^ & H | y = 0.8739x - 0.04879 | 0.6136 | 25/28 (89.29%) | -0.6736, 1.872 | 0.5993 | < 0.001 |
| G^*^ & I | y = 0.8696x + 0.1665 | 0.7796 | 26/28 (92.86%) | -0.4651, 1.27 | 0.4025 | < 0.001 |
| G^*^ & J | y = 0.8217x + 0.7460 | 0.7241 | 26/28 (92.86%) | -0.9389, 1.003 | 0.0321 | < 0.001 |
| G^*^ & K | y = 0.7587x + 1.283 | 0.5921 | 26/28 (92.86%) | -1.447, 0.9881 | -0.2296 | < 0.001 |
| G^*^ & L | y = 0.7790x + 0.9369 | 0.7261 | 25/28 (89.29%) | -0.9242, 0.9792 | 0.0275 | < 0.001 |
| H & I | y = 0.7509x + 1.135 | 0.7233 | 26/28 (92.86%) | -1.258, 0.8649 | -0.1968 | < 0.001 |
| H & J | y = 0.6974x + 1.706 | 0.6492 | 27/28 (96.43%) | -1.766, 0.6322 | -0.5671 | < 0.001 |
| H & K | y = 0.6899x + 1.996 | 0.6095 | 26/28 (92.86%) | -2.105, 0.4470 | -0.8289 | < 0.001 |
| H & L | y = 0.6577x + 1.861 | 0.6441 | 25/28 (89.29%) | -1.774, 0.6305 | -0.5718 | < 0.001 |
| I & J | y = 0.9153x + 0.7059 | 0.8715 | 26/28 (92.86%) | -1.013, 0.2726 | -0.3704 | < 0.001 |
| I & K | y = 0.9323x + 0.8529 | 0.8338 | 26/28 (92.86%) | -1.218, -0.04629 | -0.6321 | < 0.001 |
| I & L | y = 0.8487x + 0.9310 | 0.8036 | 27/28 (96.43%) | -0.9023, 0.1523 | -0.3750 | < 0.001 |
| J & K | y = 0.9323x + 0.5550 | 0.8339 | 26/28 (92.86%) | -0.9969, 0.4733 | -0.2618 | < 0.001 |
| J & L | y = 0.8490x + 0.6588 | 0.8042 | 26/28 (92.86%) | -0.7812, 0.7719 | -0.0046 | < 0.001 |
| K & L | y = 0.8657x + 0.3596 | 0.8716 | 26/28 (92.86%) | -0.3809, 0.8952 | 0.2571 | < 0.001 |

#：LOA = Limit of agreement

P: POCT = Point of care test

*: Imported HIV-1 VL kit
